# Investigation of a recent rise of dual amantadine-resistance mutations in the influenza A M2 sequence

**DOI:** 10.1186/1471-2156-16-S2-S3

**Published:** 2015-04-23

**Authors:** Matthew G Durrant, Dennis L Eggett, David D Busath

**Affiliations:** 1 Department of Physiology and Developmental Biology, Brigham Young University, Provo, UT 84097 USA; 2 Department of Statistics, Brigham Young University, Provo, UT 84097 USA

## Abstract

**Background:**

The S31N amantadine-resistance mutation in the influenza A M2 sequence currently occurs more frequently in nature than the S31 wild type. Overcoming the resistance of the S31N mutation is the primary focus of M2 researchers who aim to develop novel antiviral therapies. Recent studies have noted a possible rise in frequency of the V27A/S31N double amantadine-resistance mutation in recent years. The purpose of this study is to investigate this recent rise in frequency of the double mutation and any possible bias of the other mutations toward co-occurrence with S31N or S31 strains.

**Results:**

The primary dataset used for this study was comprised of 24,152 influenza A M2 channel sequences which were downloaded from UniProt. There is an increased frequency for the S31N/V27A dual AR mutation in recent years, especially in swine. A test for difference in two proportions indicates that the V27A mutation is co-occurring with S31N more often than expected (p-value < 0.001) when considering individual amino acid frequencies. At the same time, the different propensities for the V27A as compared to the V27T dual mutant may reflect differences in viral fitness or protein energetics, and this information could be exploited to focus drug development so as to reduce further drug insensitivity.

**Conclusions:**

The development of the S31N/V27A variant in the Midwestern US swine may be a harbinger of novel human strain development. V27A/S31N is a possible path forward for the evolution of M2 which may convey a new level of drug resistance and should receive attention in drug design.

## Background

The amantadine-resistance (AR) S31N influenza A M2 channel mutation is currently the most prevalent form of the M gene in human isolates in both H1N1 [1] and H3N2 [2] subtypes. Overcoming the AR of the S31N mutation is the primary focus of M2 channel researchers [3,4]. In addition to S31N, previous studies have shown that a number of mutations near the drug-binding site can lead to AR, including V27A, V27T, V27S, A30T, G34E and L26F [5]. It is in the interest of all researchers who are investigating the M2 channel to not only overcome S31N AR strains, but also to predict the rise in frequency of other AR mutations, including double AR mutations. The presence of double AR mutant strains was noticed in small frequencies in 2009 [6,7], and was later confirmed in 2012 by Garcia et al., who made note of a possible rise in frequency since 2009 [8]. One study conducted by Abed et al. helped to characterize the V27A/S31N mutation. Through a reverse genetics approach, these researchers produced a V27A/S31N double mutant (among other AR mutations) from the A/WSN/33 strain. They then infected 16 mice and recorded that "all mutants were at least as virulent as the WT in experimentally infected mice, with the highest mortality rate being obtained with the recombinant harboring a double V27A/S31N mutation [9]." Interestingly, however, the study also reported that the V27A/S31N double mutant is less resistant to amantadine (although still considered amantadine resistant on the whole) than either of the S31N or V27A mutations, providing some evidence against a synergistic effect of these two mutations on amantadine sensitivity.

It became clear throughout the course of this study that the V27A/S31N double mutation is rising in proportion primarily in the swine population. This raises the question of the potential for virus transmission from swine to humans. Past research has shown that swine to human transmission of the influenza A virus is possible and occurs regularly. In recent years more work has been done to more precisely trace these interspecies transmissions of the virus, and to then determine the likelihood of human to human viral transmission [10]. The swine population, termed by others as a "mixing vessel" for influenza viruses, has generally been considered the last step taken by novel influenza viruses prior to complete transmission to the human species [11]. These studies further prompt researchers to consider M2 evolutionary trends even in non-human species when designing and testing potential drugs.

These observations prompted the researchers of this study to better characterize this recent rise in dual AR mutation frequency. In the past decade, sequencing technology has improved dramatically and produced a wealth of influenza A M2 channel sequence data that enables researchers to track and predict the evolution of the virus with great precision [6]. This paper reports the utilization of a Z test for difference in proportions to characterize the frequencies of dual AR mutations using M2 sequences from the UniProt database.

## Methods

### M2 sequence set preparation

28,042 influenza A M2 protein sequences with 97 total amino acids were downloaded on August 28^th^, 2014 from the UniProt.org database (Additional file 1). This initial set was first filtered by removing experimental strain crosses, fragments, sequences with no given subtype, and otherwise poorly cataloged sequences, resulting in a set of 24,152 sequences

This initial set of all sequences was further divided into 6 subsets, three divided by isolation year, pre-2000, 2000-2010 and post-2010; and three sets divided by host organism: human, swine, and other. These subsets were further reduced by including only one copy of each unique sequence (to reduce heritance and resampling biases) that occurred at least twice (to eliminate eroneous sequence determinations) in each sequence subset. The results of applying these filters are given in Table 1. This filtering technique allows for the same unique sequence to be shared among sequence subsets in the event that it occurs in more than one subset. Each sequence subset was reduced by 95% on average, with the greatest reduction being that of the human set, an indication of especially high sequence repetition in this subset. Filtering was performed using custom python scripts. By analyzing only all unique sequences with a minimum duplicate count of two, the sequence subsets avoid two extremes in large-scale sequence analysis: analyzing all duplicates together which could potentially lead to a very strong sampling bias, and analyzing only unique sequences which could give sequencing errors much greater weight in the set. These refined sequence subsets were the primary focus of statistical analysis in this study.

**Table 1:** The effect of applying filters to sample on overall sample size.

	Total	Unique	Unique N > 2	**% Red**.
All Sequences	24152	2530	1007	95.83%

Pre-2000	2396	475	184	92.32%
2000-2010	13753	1473	560	95.93%
Post-2010	8003	909	373	95.34%

Human	13315	783	310	97.67%
Swine	3288	742	277	91.58%
Other	7549	1103	453	94.00%

Six subsets of the total sequence set were constructed: three divided by date of isolation (Pre-2000, 2000-2010, and Post-2010), and three divided by host organism (human, swine, other). Two filters were applied to the unfiltered sequence subsets ('Total'): the first filter including only unique strains ('Unique' column), and a second filter including only those unique strains that occur at least twice in the unfiltered sequence set ('Unique n > 2'). The '% Red.' column refers to the reduction in set size that takes place between the 'Total' column and the 'Unique n > 2' column. A higher percent reduction is an indication of high sequence repetition in the initial set.

### Amino acid frequency calculation

The frequency of occurrence of a given amino acid in a sequence subset was calculated by dividing the count for that amino acid by the number of sequences:

(1) $$f_{ap}=\frac{N_{ap}}{N_{t}}$$

Here, *f_ap _*is the frequency of occurrence of amino acid *a *at position *p*, *N_ap _*is the count of sequences having amino acid *a *at position *p*, and *N_t _*is the number of sequences in the subset.

### Test for the difference in two proportions: Independence from the identity of residue 31?

A test for the difference in two proportions was performed for each subset on all non-residue 31 amino acids, focusing primarily on the other amantadine-resistance cites, that occurred in at least one unique sequence to test for a significant difference in amino acid frequency in S31 and S31N strains (Additional file 2). The Z score was obtained as follows:

(2) $$Z_{ap}=\frac{\left(N_{ap,S31}/N_{S31}\right)-\left(N_{ap,S31N}/N_{S31N}\right)}{\surd \left(\left(\frac{N_{ap,S31}+N_{ap,S31N}}{N_{t}}\right)\left(1-\frac{N_{ap,S31}+N_{ap,S31N}}{N_{t}}\right)\left(\frac{1}{N_{S31}}+\frac{1}{N_{S31N}}\right)\right)}$$

Where *Z_ap _*is the Z score of amino acid *a *at position *p *(where *p *≠ 31), *N*_*ap*,*S*31 _is the count of sequences in which amino acid *a *at position *p *occurs simultaneously with (WT), *N*_*S*31 _is the count of sequences in which occurs, *N*_*ap*,*S*31*N *_is the count of sequences in which amino acid *a *at position *p *occurs simultaneously with, *N*_*S*31*N *_is the count of sequences in which occurs, and *N_t _*is the total number of sequences in the sequence subset. P-values were directly calculated from the Z scores assuming Z to be normally distributed, to evaluate the statistical significance of deviations from expected proportions. Deviations from the expected proportions are assumed, based on the reduction in heritance bias by the filtering, to be an indication that the amino acids in question are occurring preferentially with S31 or S31N due to energetic/functional effects on the fitness of the virus. A pseudo-Bonferonni corrected p-value threshold, 0.005, was applied to determine signficant deviations from expected proportions, and less than 10 unique occurences of a given amino acid was considered insufficient data to determine a bias toward S31 or S31N.

### S31N, V27A, V27T mutation frequencies over time

To evaluate the advancement of the three most common AR mutations and correlations between residues 27 and 31 (which are spatial neighbors), the unfiltered sequence sets representing strains collected from all host organisms, only humans, swine, and all other hosts were subdivided chronologically by decade of virus isolation, starting with the decade beginning in 1930 and ending with the decade beginning in 1990, and then by year starting with 2000 and ending with 2012, and finally sequences isolated between 2013 and the time the sequences were downloaded (August 2014). These sequences subdivided by year were then filtered to unique sequences occurring at least twice in the unfiltered set, as described previously.

The frequency of the amino acids S31N, V27A and V27T in each individual subset was calculated according to equation 1.

Also, the proportion of S31N mutants occur simultaneously with V27A or V27T was calculated as:

(3) $$f_{dm}=\frac{N_{27A/N31}}{N_{N31}}$$

(4) $$f_{dm}=\frac{N_{27T/N31}}{N_{N31}}$$

Where *f_dm _*is the frequency of double mutants within the S31N sample, *N*_27*A*/*N*31 _is the count of sequences that possess both a V27A and an S31N mutation, *N*_27*T*/*N*31 _is the count of sequences which possess both a V27T and an S31N mutation, and *N*_*N*31 _is the count of sequences which possess an S31N mutation. 95% confidence intervals of the frequencies were calculated for each subset using a 1000x bootstrapping technique, implemented using the boot package (v1.3-13) in R. Each subset was resampled with replacement to produce 1000 subsets of equal size to the original, and the frequencies of the amino acids of interest were calculated for each to produce a distribution that was then analyzed by the boot.ci function to produce 95% confidence intervals.

## Results

### General details of overall sequence set

The overall sample of 24,152 sequences was comprised of strains collected from ~266 different organisms (Additional file 2), which were grouped as human (55.3%), swine (13.6%), and other organisms/sources (31.1%). The sample was comprised of 82 different influenza subtypes in total: 37.4% H1N1, 28.4% H3N2, 6.0% H5N1, 3.9% H1N2, 3.8% H3N8, and 20.5% other subtypes (Additional file 2). Approximately 887 different locations are represented in the sample (Additional file 2), 530 of which are associated with two or more sequences, and 54 are associated with 100 or more sequences. The 54 most represented locations are associated with 68% of all sequences. Of these (16,434 sequences), 65.72% were collected in North America, 12.24% in Asia, 8.95% in Europe, 8.25% in Southeast Asia, 4.17% in South America, and 0.66% in Africa.

### Amino acid frequencies indicate highly conserved regions and evolutionary trends

**Figure 1:**
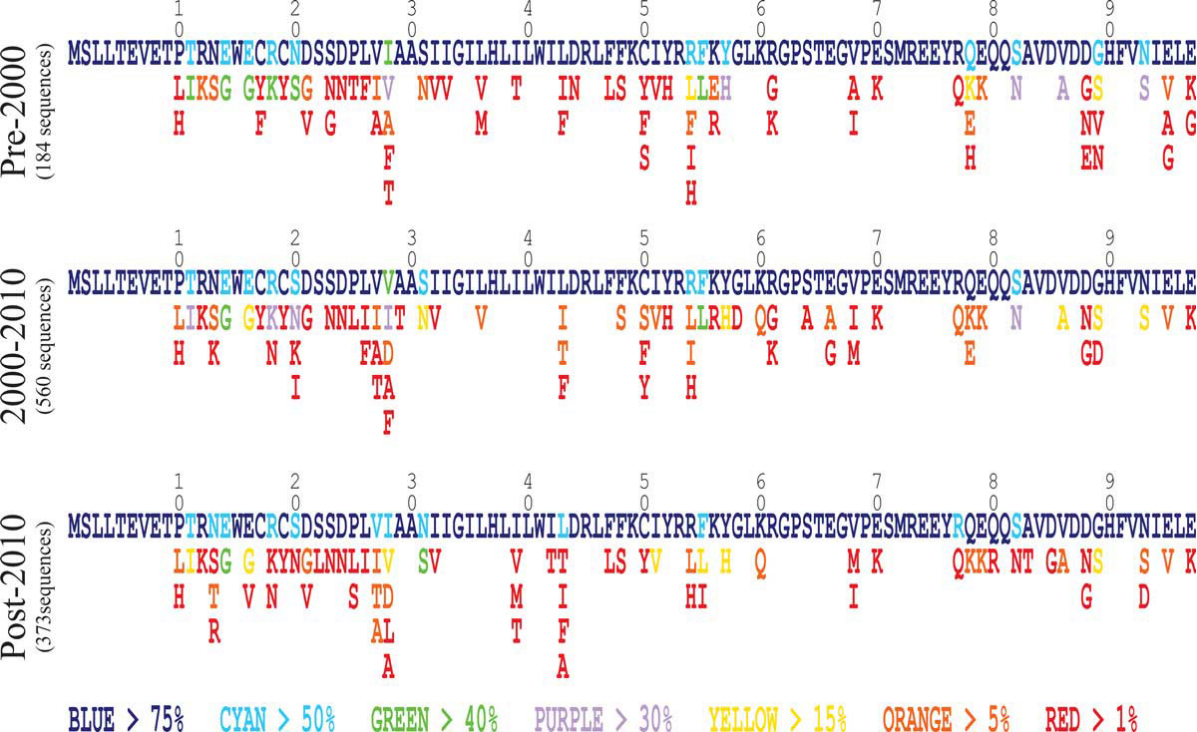
**General evolutionary trends of the Influenza A M2 Channel over three epochs**. Frequencies of each amino acid occuring in more than 1% of strains are shown for the three epochs: previous century, past decade, and current decade. This table does not filter out specific host organisms; they are all considered together. Colors are assigned according to amino acid frequency; if the percentage of occurrences for an amino acid within the entire set is between two of the frequency cutoffs (specified in the legend below), it is assigned the color associated with the lower cutoff.

In order to better understand general evolutionary trends for the M2 protein, an amino acid frequency table was generated for sequenced isolates dated 1933-2000 (pre-2000), 2000-2010, and 2010-present, (Additional file 2). Dominant sequences and deviations in the filtered subsets (unique sequences occuring more than once) for these three time periods are shown in Figure 1. The results indicate the protein domains which are evolutionarily conserved, as seen by a general lack of amino acid diversity in specific regions. Residues 1-10 of the N-terminal domain are highly conserved, as are several residues of the transmembrane domain (residues 22-49), especially His37 which forms the selectivity filter. In spite of considerable sequence fluctuation within eras, the level and breadth of diversity is very steady over the three different eras, indicating a high level of functional/energetic requirements at each position, consistent with the well-defined structure of the entire protein [12-14] and the numerous specific functions of each domain in the protein [15-19]. Variations that appear in more than 1% of the sequences are generally of the same side chain type and never exceed four in number. Only at 18 positions (11, 13, 14, 15, 18, 20, 27, 28, 31, 43, 54, 55, 57, 77, 78 82, 89 and 93) do the dominant residue frequencies drop below 75% occurrence frequency in any of the three groups, and even these sites generally have homologous substitutions. Only at positions 11, 14, 16, 18, 20, 28, 31, 55, 57, 82, 86, and 93 do any of the variants from the dominant residue ever reach a frequency >30%, and in all of these cases only a single substitution is so common.

With regards to AR mutations, the dominant amino acid at position 31 shifts from 71.6% serine (S) to 59.9% asparagine (N) between the 2000-2010 and post-2010 sequence sets, as reported previously [1,2]. The AR V27A mutation rises in prevalence from 1.1% to 7.8% of unique sequences between sets 1 and 3. V27T follows a similar trend to V27A, but with a yet lower frequency (0.5%) than V27A in the pre-2010 sequence set, and a yet higher frequency (8.8%) in the post-2010 sequence set. V27S is a rare mutation, never appearing with a frequency greater than 1%. L26F occurs with frequency greater than 1% only in the pre-2000 and 2000-2010 sequence sets. A30T and G34E occur with <1% (or never) in all three filtered sequence sets. Position 28 is not a recognized AR site but has been recently found to modulate the impact of residue 31 on drug blocking (personal communication, David Fedida and Ian Tietjen). It shows a shift in amino acid frequency across the three sequence sets, with isoleucine (I) being the most frequent amino acid in the pre-2000 sequence set, valine (V) being the most frequent in the 2000-2010 sequence set, and a reversion to isoleucine in the post-2010 sequence set.

In summary, AR mutations S31N, V27A, V27T, and L26F all occur in significant numbers in at least one of the three sequence sets. AR mutations V27S, A30T, and G34E do not occur with significant frequency. In the most recent period, the post-2010 sequence set, S31N, V27A, and V27T are the only three AR mutants that appear with significant frequency. No variation other than N has appeared (>1%) in isolates at position 31. V28I has increased and may cause further complications for M2 researchers.

### Test for difference in proportions indicates a significant V27A/S31N double AR mutation bias

A test for difference in proportions was performed to indicate whether each non-S31N AR mutation disproportionately occurred in conjunction with S31 or S31N for the six subgroups. In addition, the frequencies of mutation from proline to leucine or histidine at position 10 were evaluated for the S31 and S31N groups as a control due to the expected spatial separation of residue 10 from residue 31, and an assumed lack of functional relations between them that might produce evolutionary pressure.

Between the three epochal subsets, the apparent bias of each of the non-31 AR mutations toward S31 or S31N is shown in Table 2. Prior to year 2000, the unique, replicable sequences with S31N were sparse (N_S31N _= 12), so the statistics are not persuasive, but AR mutations V27A, V27T, and appear disproportionately in conjunction with the S31N AR mutation and no preference for S31 can be demonstrated. Between years 2000 and 2010, V27A appears with both S31 and S31N strains, but V27T dominates with S31 strains. Following 2010, V27A heavily favors S31N, occurring in conjunction with S31N in 29 sequences, while never occurring in conjunction with S31. V27T shows the opposite pattern, occurring in conjunction with S31 a total of 33 times, and never with S31N. V28I appears in conjunction with S31N more often than expected.

**Table 2:** Test for difference in proportion table for sequence sets divided date of isolatiion

	Pre-2000			
	N_t _= 184, N_S31 _= 171, N_S31N _= 12			
	
	AA+S31	AA+S31N	Favors	P-value
10L	6	0	NA	ID
10H	4	0	NA	ID
26F	1	1	NA	ID
27A	0	2	NA	ID
27T	0	1	NA	ID
27S	0	0	NA	ID
27I	21	0	NA	0.1
28I	71	11	**S31N**	**<0.001**
30T	0	0	NA	ID
34E	0	0	NA	ID

	**2000-2010**			
	N_t _= 560, N_S31 _= 401, N_S31N _= 157			
	
	AA+S31	AA+S31N	Favors	P-value

10L	31	9	NA	0.2
10H	13	7	NA	0.24
26F	2	5	NA	ID
27A	11	7	NA	0.15
27T	8	0	NA	ID
27S	2	0	NA	ID
27I	69	4	**S31**	**<0.001**
28I	154	57	NA	0.32
30T	1	0	NA	ID
34E	0	0	NA	ID

	**2010-present**			
	N_t _= 373, N_S31_=151, N_S31N _= 222			
	
	AA+S31	AA+S31N	Favors	P-value

10L	8	13	NA	0.41
10H	8	8	NA	0.21
26F	0	3	NA	ID
27A	0	29	**S31N**	**<0.001**
27T	33	0	**S31**	**<0.001**
27S	0	0	NA	ID
27I	33	4	**S31**	**<0.001**
28I	73	133	S31N	<0.05
30T	0	0	NA	ID
34E	0	0	NA	ID

The amino acids of interest are included in the left column. The 'AA+S31' column is the count of amino acids in the left column that occur in conjunction with S31 (WT). The 'AA+S31N' column is the count of amino acids in the left column that occur in conjunction with S31N. The 'Favors' column indicates the amino acid, S31 or S31N, with which the amino acid in the left column occurs more often then expected, according the test for difference in proportions. The 'P-value' column gives the p-value of each test for difference in proportion. ID: insufficient data (<10 samples of the AA).

To evaluate whether the results are robust, as opposed to (for example) reflecting host species biases for certain viral strains, the sequence set was divided into sets of human, swine, and all other hosts rather than epochs (Table 3). Among dual AR mutations, V27A/S31N is most common in swine hosts. Human sequences seem to be largely void of double mutations, as do the other host sequences. However, the control mutation P10L shows a disproportionate bias toward S31N possibly indicating a strong heritance bias in the swine set.

**Table 3:** Test for difference in proportion table for sequence sets divided by host organism.

	Human			
	N_t _= 313, N_S31 _= 151, N_S31N _= 161			
	
	AA+S31	AA+S31N	Favors	P-value
10L	1	2	NA	ID
10H	1	3	NA	ID
26F	2	2	NA	ID
27A	5	6	NA	0.42
27T	0	1	NA	ID
27S	0	0	NA	ID
27I	3	2	NA	ID
28I	11	74	**S31N**	**<0.001**
30T	0	0	NA	ID
34E	0	0	NA	ID

	**Swine**			
	N_t _= 277, N_S31 _= 145, N_S31N _= 131			
	
	AA+S31	AA+S31N	Favors	P-value

10L	1	5	NA	ID
10H	5	4	NA	ID
26F	0	4	NA	ID
27A	1	29	**S31N**	**<0.001**
27T	38	0	**S31**	**<0.001**
27S	2	0	NA	ID
27I	69	4	**S31**	**<0.001**
28I	6	111	**S31N**	**<0.001**
30T	0	0	NA	ID
34E	0	0	NA	ID

	**Other Hosts**			
	N_t _= 539, N_S31 _= 447, N_S31N _= 91			
	
	AA+S31	AA+S31N	Favors	P-value

10L	48	18	S31N	**<0.01**
10H	22	8	NA	0.21
26F	1	1	NA	ID
27A	8	4	**NA**	**<0.001**
27T	5	0	**NA**	**ID**
27S	0	0	NA	ID
27I	50	0	**S31**	**<0.001**
28I	274	22	**S31**	**<0.001**
30T	1	0	NA	ID
34E	0	0	NA	ID

The amino acids of interest are included in the left column. The 'AA+S31' column is the count of amino acids in the left column that occur in conjunction with S31 (WT). The 'AA+S31N' column is the count of amino acids in the left column that occur in conjunction with S31N. The 'Favors' column indicates the amino acid, S31 or S31N, with which the amino acid in the left column occurs more often then expected, according the test for difference in proportions. The 'P-value' column gives the p-value of each test for difference in proportion. ID: insufficient data (<10 samples of the AA).

In summary, in recent years the AR V27A mutation is occurring in conjunction with S31N more frequently than expected by chance, while V27T is occurring primarily with S31. Both of these mutations are primarily occurring in swine-host strains. Proline variants H and L at the presumably nondiscriminatory position 10, were insensitive (P > 0.005, with the pseudo-Bonferroni correction) to the residue at position 31 in 5 of the 12 cases tested, but were sensitive in 1 of the 12 cases tested, suggesting a possible heritance bias. (Data was inadequate in 6). Therefore, it is not certain from this test whether or not the bias that amino acids such as V27A have toward S31N is due to a functional or energetic advantage as opposed to heritance effects. Nevertheless, with the higher numbers of sequences representing the V27A, V27T, and S31N mutations, it seems likely that functional and/or energetic effects are important and it is clear that the V27A/S31N dual AR mutation is occurring more often than expected by chance.

### Year by year frequency of S31N, V27T, and V27A mutations shows distinct rise in double mutations

**Figure 2:**
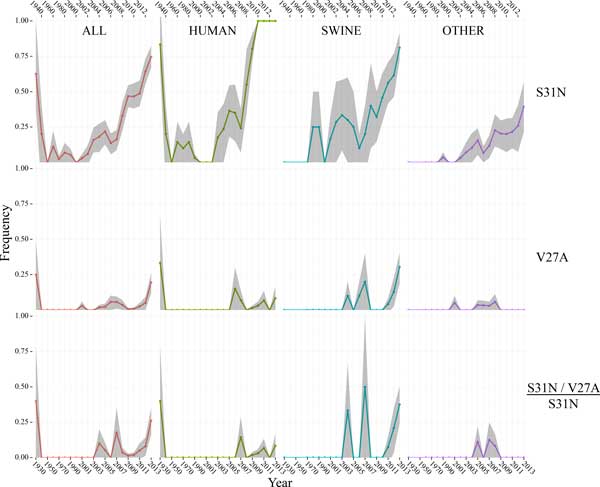
**V27A and S31N mutations over time**. Frequencies of mutation appearances relative to the total number of sequences in the subgroup (rows 1 and 2) or the total number of S31N mutants in the subgroup (row 3). Columns of panels are labelled according to the host organism associated with each sequence subset. 95% confidence intervals are indicate the grey ribbon. Years are indicated both on the bottom and the top of the figure.

**Figure 3:**
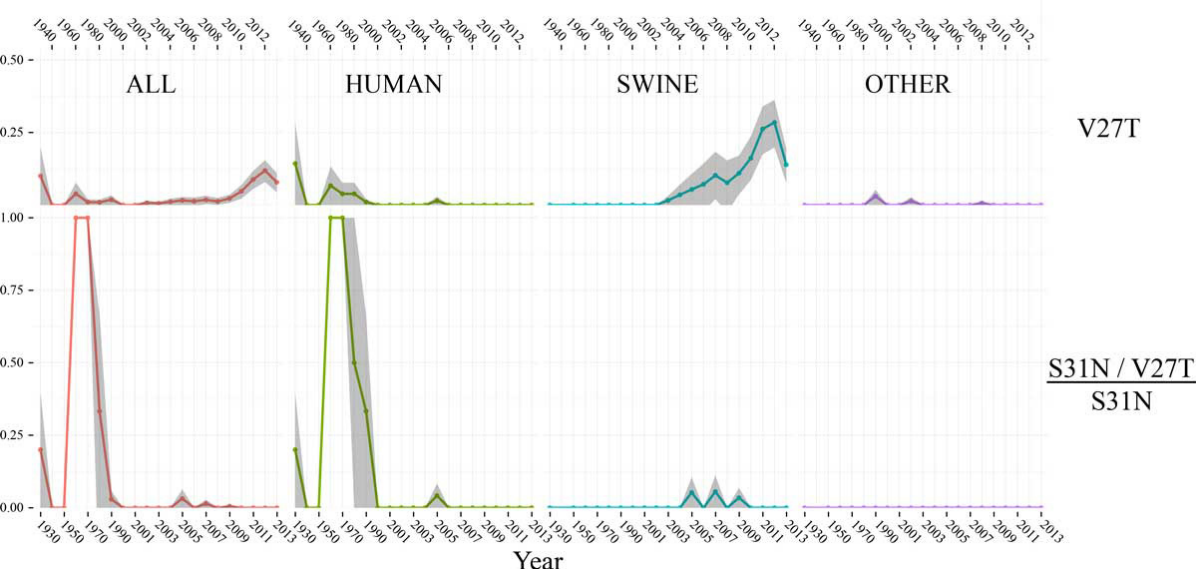
**V27T mutations over time**. Columns are labelled according to the host organism associated with each sequence subset. 95% confidence intervals are indicate the grey ribbon. No confidence intervals are shown for the 1950 point (second row of panels), which represent only one case of human isolate. Years are indicated both on the bottom and the top of the figure.

In order to better correlate and visualize the changes in S31N, V27A (Figure 2), and V27T (Figure 3) mutation frequencies with greater time resolution, the relative frequency of each amino acid over time was calculated as described in the methods section. In the 1930s and the 1940s, a higher frequency of S31N mutations (Figure 2, top row of panels) is evident as a result of the heavy sampling bias toward A/WSN/1933, a strain of great interest to researchers due to its S31N mutation and the fact that it was the first isolated influenza strain. The large 95% confidence intervals indicate that the high number of S31N mutations in this sample set is not significant due to the small sample size. However, from year 2005 on there is a steady increase in S31N mutation frequency within all species considered together. In the human host strains, the S31N mutation is present in 100% of sequences from the year 2010 on. Somewhat unexpectedly, the S31N mutation is not quite as prevalent in the swine subset, comprising ~80% of all swine sequences in the most recent year. The S31N mutation has also increased in frequency in other hosts but not to the same extent as in human hosts or swine hosts, reaching ~40% of all other host sequences in the most recent year.

V27A (Figure 2, 2^nd ^row of panels) has increased in frequency among swine in recent years, rising from 0% in 2010 to ~30% in the most recent year. Earlier occurrences of the V27A mutation do not appear to be significant according to the 95% confidence intervals. Additionally, the V27A/S31N double AR mutation (Figure 2, 3^rd ^row of panels) is occurring more frequently in recent years, with the V27A occurring in ~40% of S31N swine-host strains. The V27T mutation, which was shown in the previous section to occur primarily with S31 over S31N, has also increased steadily in frequency among swine (Figure 3, first row of panels), but in the most recent year it did decline in frequency from ~26% to 15% of all swine strains. It only occurs in significant numbers in the swine sample. In contrast to V27A, the V27T mutation did not increase significantly in frequency among S31N strains (Figure 3, second row of panels, especially in the swine column) as its overall frequency increased. This provides further evidence that there is a distinct bias for the V27T AR mutation to occur in conjunction with S31, and not with S31N. This analysis identifies a distinct correlation between positions 27 and 31.

### Details of dual AR V27A/S31N mutants

A total of 553 double AR V27A/S31N M2 total sequences were collected from swine (of which 29 sequences were unique and occurred at least twice). Of the 553 V27A/S31N sequences, 135 were collected from swine in Ohio, 80 from Iowa, 78 from Indiana, 75 from North Carolina, 54 from Illinois, 27 from Minnesota, 15 from Korea, 13 from Nebraska, 10 from Kansas, and the remaining 54 were shared among 27 other unique locations, primarily in the Midwestern United States. The large sampling of swine from Ohio is due in part to an active surveillance project being carried out by researchers at Ohio State University [10]. 532 of these strains have been collected since 2009, and 284 were collected in 2013 and 2014. 285 sequences belong to the H3N2 subtype, 148 belong to H1N2, 117 sequences belong to H1N1, and 3 belong to H3N1.

Only 42 dual AR V27A/S31N sequences (total) have been collected from human hosts. 10 of these sequences belong to the A/Puerto Rico/8/1934 sequence, which has been repeatedly sequenced. Of the remaining 32, 5 were collected in Indonesia, 4 in Australia, 3 in Iowa, 3 in Tennessee, 2 in Helsinki, and the remaining 15 were collected from 13 other locations. 19 of the remaining 32 have been collected since 2009, and 4 have been collected in 2013 and 2014.

Thus the V27A/S31N dual-AR M2 sequence is very common in Midwestern swine, and although it appears in human hosts at only small relative frequencies, it is broadspread geographically.

## Conclusions

The results of this study should function as a starting point for further investigation into the potential risk presented by double AR mutations. V27A/S31N double AR strains are presenting themselves more frequently in swine in recent years. Similarly, V27T appears more frequently in swine than other species, but with S31. Even upon further sample filtering to analyze only influenza strains isolated from swine in the USA, the bias of the V27A mutation toward S31N strains is still strongly significant (p-value < 0.001), with no V27A/WT unique M2 sequences and 26 unique V27A/S31N M2 sequences occurring in this subset. It is possible that the proton transport properties of the M2 channel that depend on luminal residues 27 and 31 co-vary, with the small (methyl) side-chain of A27 allowing more space for water and protons to enter and interact with the polar N31 side chain, whereas the V27T mutation more subtly enhances water entry through polarity in the side chains, somehow more consonant with the mild polarity of S31 side chains.

The possibility of a second viable amantadine resistant mutation could further complicate the endeavors of Influenza M2 channel researchers, potentially rendering novel S31N blockers ineffective. Future anti-viral studies should explore blocking effects in both S31N and V27A/S31N mutants.

## Competing interests

The authors declare that they have no competing interests.

## Authors' contributions

MGD designed the study, collected the data, conducted the analyses, and drafted the manuscript. DLE determined the proper use of the Z test for difference in proportion and was a general consultant in regards to all the statistical analyses performed in the study. DDB participated in the design of the study, interpretation of the results, and drafting the manuscript.

## Supplementary Material

Additional file 1 **Raw Sequence Data**. This is a fasta protein sequence file. It contains the unfiltered M2 sequence data as it was originally downloaded from the UniProt database. Click here for file

Additional file 2 **Supplementary Data**. This is an Excel spreadsheet file (.xlsb) The content of this file are as follows: Supplementary Table 1: A table demonstrating the proportion of all non-human host organisms from the original sample of 24,152 sequences. Supplementary Table 2: A table demonstrating the proportion of the various subtypes from the original sample of 24,152 sequences. Supplementary Table 3: A table demonstrating the proportion of each strain collection location from the original sample of 24,152 sequences. The top 55 locations are also labeled by the region in which they reside, designated as follows: North America (NA), Asia (AS), Southeast Asia (SEA), South America (SA), Africa (AF), Europe (EU), and Australia (AU). Supplementary Table 4: This table contains the results of the test for difference in proportion for each amino acid that occurs at all 97 positions in all sequence subsets. The "Sequence Set" column refers to the subset to which the amino acid and subsequent data belong. There are 7 subsets represented, all of which include only unique sequences that occur at least twice: All Sequences (ALL), PRE-2000, 2000-2010, 2010-PRESENT, HUMAN, SWINE, OTHER HOSTS. The next two columns include the amino acid of interest and its position. The 'Total' column refers to the total sequence count in the subset. Count S31 and Count S31N refer to the count of each of the two amino acids in the subset. These numbers do not always add up to the Total column, because there are a small number of sequences that have an alternative mutation at position 31. Count AA+S31 and Count AA+S31N refers to the count of sequences that contain both the amino acid of interest and the S31 or S31N amino acids. The Z-score and p-value columns demonstrate whether the proportions deviate significantly from the expected distribution according to the pseudo-Bonferroni corrected p-value of 0.005, represented by green highlighted cells. In the event that the statistics indicate a significant bias toward either S31 or S31N, the amino acid is shown in the 'Favors' column. ID: insufficient data (<10 samples of the AA) NA: Not applicable, the amino acid position only had one variant in the sample. Click here for file

## Declarations

The publication costs for this article were funded by Brigham Young University.

This article has been published as part of *BMC Genetics *Volume 16 Supplement 2, 2015: Selected articles from The 11th Annual Biotechnology and Bioinformatics Symposium (BIOT-2014): Genetics. The full contents of the supplement are available online at http://www.biomedcentral.com/bmcgenet/supplements/16/S2.
